# A Perceptive Interface for Intelligent Cyber Enterprises

**DOI:** 10.3390/s19204422

**Published:** 2019-10-12

**Authors:** Ioan Dumitrache, Simona Iuliana Caramihai, Mihnea Alexandru Moisescu, Ioan Stefan Sacala, Luige Vladareanu, Dragos Repta

**Affiliations:** 1 Automatic Control and Systems Engineering Department Bucharest, University Politehnica of Bucharest, 999032 Bucharest, Romania; ioan.dumitrache@acse.pub.ro (I.D.); ioan.sacala@acse.pub.ro (I.S.S.); 2 Automatic Control and Industrial Informatics Department Bucharest, University Politehnica of Bucharest, 999032 Bucharest, Romania; simona.caramihai@aii.pub.ro (S.I.C.); repta_dragos@yahoo.com (D.R.); 3 Institute of Solid Mechanics of the Romanian Academy, 010141 Bucharest, Romania; luige.vladareanu@vipro.edu.ro

**Keywords:** cyber-physical systems, intelligent cyber enterprise, systems interface

## Abstract

Large scale, complex, networked enterprises, as may be considered (trans)national energy systems, multi-national manufacturing enterprises, smart cities a.s.o. are structures that can be characterized as systems of systems (SoS) and, as such, require specific modelling paradigms and control architectures to ensure their successful running. Their main characteristic is the necessity of solving practically one-of-a-kind problems with respect to the external context and internal configuration, thus dealing with dynamically evolving flows of data and information. The paper introduces the concept of intelligent cyber-enterprise, as an integrating paradigm that uses information and knowledge dynamics, in order to model and control SoS, especially focusing on the importance of appropriately adapt external and internal perception of an enterprise through a new generation of sensorial systems—the perceptive interfaces. The authors analyze sensing and perception in relation to intelligent cyber enterprise model and propose an implementation for a perceptive system interface.

## 1. Introduction

New technological developments have helped solve many scientific and socio-economic problems over the years. Nowadays we can produce goods in every economic sector, and we can transport them in very short time no matter what distance is required all around the globe. This can be done nowadays much faster than ever. We can extract the required data from very large amounts of data, found in a single location or combine from multiple locations through heterogenous interconnected networks. We may use the information provided in a very short period, so that we may easily find a solution and adapt depending on the circumstances.

Availability of resources, personalized products, social enterprise, environmental awareness, globalization of markets, all of these represent important factors in today’s enterprises’ management and economic development. The problem is, they represent also pressure factors in the decision-making process, considering the following aspects:

- Decision making is often a problem of selecting from several possible solutions, based on available information and optimization (efficiency) criteria.
- As now there are huge amounts of available data, this availability often increases the complexity of the problem, by taking into account too much information (irrelevant for the core of the decision).
- This complexity either may generate such models that their analysis and performance evaluation take too much time with respect to the problem-solving time horizon or may encourage model simplification (including finding false similarities) which results in incorrect models.

Either way, a correct decision making implies both an appropriate problem modelling and a correct selection of relevant information and data to be used (gathered and exchanged) in its solving.

The approach of this paper is thus oriented on:

- Defining a modelling approach for a system of systems structure, oriented on problem-solving (the intelligent cyber-enterprise).
- Defining a generic problem model, in order to support systems dynamics, reconfiguration and adaptation to the environment and context, with respect to information flow (the perception-reasoning-learning model).
- Identification of the role and place of perceptive interfaces in ensuring the appropriate gathering and flow of information (including human in the loop aspect).

Some aspects related to process identification in order to allow appropriate design of perceptive interfaces are included. Information technologies that are used in control systems and knowledge management represent the most important aspects out of all the tools that are available on the market. Also, because the degree of complexity has increased in different engineering domains, new models and paradigms appeared over the years. On the other hand, globalization and sustainable development require a new economic perspective that must include social and environmental impact in order to develop sustainable businesses and production systems [1]. In the present paper the authors analyze sensing and perception in relation to the intelligent cyber enterprise model and propose a new concept: perceptive system interface.

## 2. Related Work

New emerging concepts, such as internet of things (IoT) and cyber-physical systems (CPS) paradigm represent only a small part of the technological drivers of the next industrial revolution. In this section we address important concepts and paradigms related to the development of intelligent cyber enterprise model.

CPS are related to the study of complex systems as well as systems of systems focusing on physical and virtual components, interactions, process interconnections and information processing and addressing a wide range of temporal and spatial scales [2]. CPS are characterized by the National Science Foundation [3] as “engineered systems that are built from and depend upon the synergy of computational and physical components. Emerging CPS will be coordinated, distributed, and connected, and must be robust and responsive.” 

Cyber-physical systems must have the capability to be reconfigurable in order to integrate the human factor into system engineering and socio-technical systems. This new trend emphasizes the constant need of interoperability paradigm, seen both from the classical considerations as well as the new trends as we must sense and perceive the smart system taking into consideration all interoperating systems. This new shift can have an important consequence in the near future in the design process as well as the implementation phase. The adoption of cloud-based technologies will have an impact on the design and implementation on the next generation of sensing systems [4].

With the development of the internet of things and future internet paradigms, the number of systems that need to cooperate and interact in the future will increase both in number and complexity. Thus, we must perceive the purpose of every system in order to use the proper methodology in order to verify and validate each functionality. One solution can be the model-based cyber-physical systems [5].

Advances in internet-oriented paradigms have led to the development of Edge/Fog computing model aiming to optimize smart applications to expand their functionalities close to the geographic location of the IoT devices, rather than outsourcing computations to far away datacenters. A proposed architecture includes an edge/fog/cloud monitoring system and a capillary container orchestrator that is able to handle highly dynamic IoT environments in which the edge node may be overloaded at runtime due to an increase in the workload [6].

Security is an important component of future internet systems. One important aspect in addressing security is trust. Edge, fog and cloud computing have to rely on resources and services under ownership of various entities [7]. A proposed trust management architecture that relies on the use of blockchain-based smart contracts (SCs) and specifically designed trustless smart oracles is presented in [7].

Correlated with these concepts, new enterprise models have emerged. Such models have been related to the factories of the future paradigm. Factories of the future aggregate the concepts and represent the future of an enterprise that is built on collaboration, connectivity at different organizational structures, machineries and human operators, in order to become part of interconnected networks of suppliers, transporters and customers. 

Distributed manufacturing systems are an important component of factories of the future and require new scheduling optimization models. A proposed model is presented in [8] and is developed based on a discrete fruit fly optimization algorithm with three heuristic methods proposed to initialize.

Another important component of factories of the future is represented by material flow analysis. Results have been used with success in optimizing material flows and waste streams in production processes [9].

A sensing enterprise represents a new paradigm based on the digital innovation concept that deals with the adoption of sensing and future internet technologies. Sensing enterprises are mostly connected to virtual enterprises and their networks. Seen from the perspective of a smart system in general, sensing enterprises must have the capability to sense, model and interpret signals from the real world and thus to be able to adapt into a more agile configuration of the system [10]. In relation to such systems a product service systems conceptual framework is proposed in [11] in order to facilitate the development of interoperable product systems and service systems in accordance with the stakeholders needs. 

Another important model associated with factories of the future is the cognitive manufacturing. Cognitive manufacturing is a proposed manufacturing organization method, based on perception and cognition that integrates IoT principles, Artificial intelligence and data analytics technologies. One of the main objectives of cognitive manufacturing implementations is to integrate process as well as enterprise wide data and information, as to achieve improvements in the use of equipment, processes reengineering based on decision-making models and data analytics models, enterprise integration of knowledge management models [12].

Cognitive manufacturing systems address the following:

- Improvement of product lifecycle management by providing an integrating enterprise and environment focused approach;
- Adaptive systems integration;
- Analyzing manufacturing data obtained from sensors: production management systems are generating huge amounts of manufacturing data;
- Using decision making models in correlation with business intelligence systems;
- Linking the overall decision-making procedures of the enterprise with knowledge management systems.

## 3. Intelligent Cyber Enterprise

In this section, the authors briefly describe the concept of intelligent cyber-enterprise (ICE), [13] seen as a socio-technical complex system. In relation to the implementation enterprise wide CPS systems the concept of ICE was proposed.

The connection between physical processes and their cybernetic representation is a very important aspect in order to modeling and design exactly the behavior of a process and thus being capable to integrate hardware-in-the-loop and human-in-the-loop components [5].

A cyber-enterprise represents an aggregation of physical objects, knowledge represented through process models (workflows), control algorithms, humans and information represented by adequate software tools and communication processes [14]. Thus, within an ICE, all these components must be capable to interact and cooperate in order to achieve intelligent enterprise goals, in terms of production, costs, time bound and resources limitations in order to satisfy all clients, suppliers and economic partners in a sustainable environment. Such a proper interaction must take into consideration the level of required intelligence, the amount of required data and how to extract information from heterogeneous flows within the enterprise, as well as by the emergent intelligent behavior of the enterprise in order to become adaptive, reactive or proactive, depending on the economic constraints and demands [15].

The ICE must be modeled in order to be capable to solve any specific problem, being capable to divide any problem at the lowest level of complexity. On the other hand, problems that must be solved are goals existing at the operational level that must include the minimum amount of enterprise resources.

**Figure 1:**
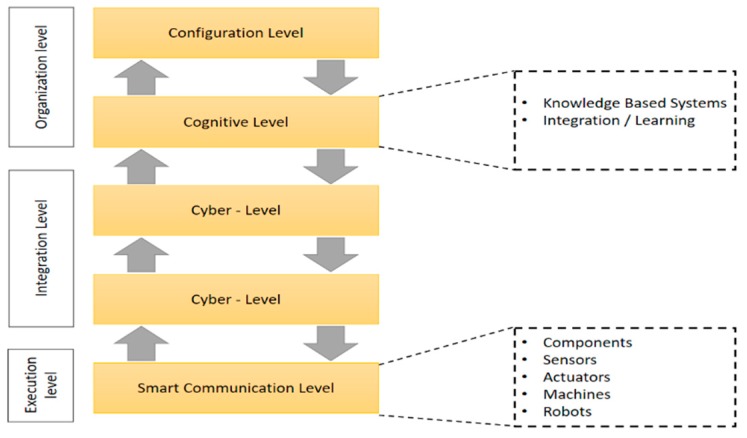
CPS-based enterprise model. Adapted from [16].

From the CPS perspective, in order to achieve these results, we must include at least a control loop and at least one enterprise resource. The more complex a problem becomes there is a demand to include multiple control loops and specific resources. Control loops must operate in real time and must be based on targeted algorithms and rules, using well defined sets of data. The more complex a problem becomes there is a necessity to integrate heterogeneous information structures and heuristics. An intelligence-based model for the ICE is represented in Figure 1.

Every component of the ICE architecture must be capable to be independent according to different pre-defined specifications and must have the capacity to communicate, allowing the transfer of meta-data, information and knowledge in order to provide reliable context-oriented behavior [17].

Another aspect that must be taken into consideration is to connect subsystems in clusters, depending on the initial functional requirements. For examples, machines are a part of manufacturing cells that communicate based on protocols, standards and rules, all of them being embedded in control algorithms. Thus, functionalities can be added afterwards depending on the required specifications, initial or during the process implementation. Manufacturing cells are part of a manufacturing system, and thus are capable to fulfill any given problem and to develop various products [3]. The main characteristics of ICE include:

- *Perception*: this characteristic has the purpose to develop and integrate results from measurement science for sensing and perception. The role of perception is to better understand the system’s complexity and thus to reduce the risks related to the adoption of new technologies.
- *Mobility of Systems*: this characteristic will develop specific test methods in order to determine the performance of each intelligent system.
- *Human-Machine Interaction*: this characteristic has the purpose of delivering specific test methods, protocols and information models in order to facilitate a more effective human-machine collaboration.
- *Agility of Industrial Systems*: this characteristic has the purpose of delivering agility performance metrics, data sets and information models in order to enable manufacturers to adapt and reconfigure system components.
- *Embodied AI and Data Generation for Manufacturing*: this characteristic has the purpose to deliver structural artificial intelligence and machine learning models and tools in order to improve the performance and autonomy of manufacturing applications.
- *Collaboration*: this characteristic has the purpose to deliver specific models in order to facilitate calibration, coordination, in order to mitigate the lack of advanced automation.
- *Safety Performance*: this characteristic will provide performance metrics, specific test methods and measurement tools in order to support the development of next-generation systems that will have the capability to integrate human behavior and to enable tactual-based safe human collaboration and manufacturing tasks.

Intelligent cyber enterprise systems design is focused on adopting new technologies in order to become agile, safe and productive, being also capable to interoperate with smart manufacturing applications and to better evolve.

### 3.1. Sensing in Correlation to the Enterprise Environment

In order to easily reconfigure, taking into consideration all existing constraints, a sensing system need different resources and conditions in order to be constantly under control. All the functions of a sensing system can be seen as a CPS. These systems can easily make the switch between physical world and thus merge to virtual world. By the help of sensor networks and actuating systems, the sensing system can monitor all the physical processes, collect data from all devices connected, interpret and expose data in different formats in order to make it widely available for every interested stakeholder and software applications to use it in order to better understand the real world.

The artificial intelligence concept can be seen from two different perspectives: both from the traditional approach but more important, from the machine learning perspective. Thus, a smart system will need to be capable of integrating generating mechanisms (physical and virtual) in order to extract the exact data that is needed. This might mean that simulation tools must be used in order to better under the behavior of the system and thus know how to instantly react based on its behaviors. Or, it might mean that data can be extracted from different web resources or multiple physical systems.

In order to develop a measurement mechanism in order to better understand a smart system, and thus sense and perceive the system performances in order to reduce the risk related to the adoption of new technologies, there is a need to integrate sensors and algorithms in order to create productive manufacturing environments.

If in the past we could identify manufacturing systems formed from simple sensors, actuators and controllers, now, there is an urgent need to integrate complex sensor networks in order to use them for the system perception. This is a complex task that makes it very difficult to implement it for academia, system integrators and even manufacturers in order to identify the right solution. The technical idea is to identify existing and emerging sensing and perception technologies and related software in order to understand the product and research solutions landscape.

Due to specific constraints related to flexibility and reusability, smart systems play a very important role in strengthening worldwide manufacturing competitiveness by stimulating responsiveness and innovation. To achieve this goal, smart systems must be capable to perceive and adapt depending on the need of collaboration with other smart systems or humans. Thus, they must have the capability to learn, adapt and quickly integrate into the rest of the enterprise.

### 3.2. Perception and Cognition in Correlation to Enterprise Environment

The design of enterprise systems based on the ICE model includes the integration of perception, reasoning, learning and cognition models. These aspects become relevant in the development of perceptive system interfaces for the ICE. An important aspect of ICE is related to extending the enterprise sensing function to a perception function. The perception-reasoning-learning loop (PRL) is further addressed, from a functional point of view and with a special emphasis on the awareness concept.

The ICE functioning is based on problem solving. Usually, such problems are described in terms of system-specific production/functioning goals, with different levels of precision, from strategical (profit versus investments, sustainability, market coverage, utilities availability, durations for problem-solving, quality of processes and products, safety, etc.) to more resource oriented, as number of products/services delivered, cost/profit per product, a.s.o.

Every problem is solved by a sequence of activities, performed by specified resources, at given times (eventually triggered by specific events)—which will denominate as workflows, and which are pieces of knowledge.

Resource oriented problems are solved by deterministic, predictable, well-defined workflows; operational and strategic problems, implying collaboration between resources, are solved by ad-hoc combination of workflows, with respect to availability of resources and context. Such problems may be, usually, solved in different ways, implying very different resources, approaches (interconnections of resources), costs and time. Solving approaches are determined mainly by resource availability, but also by the information availability, interpretation and possibility of gathering (sensing/perception). Also, networking is determined by the possibility of information interoperability between resources.

Appropriate interfaces for information transmission and appropriate perception of external information become crucial for such problem-solving approach. As designing specific interfaces for any possible problem is impossible, it is important to focus on the interface adaptability in order to allow the maximum flexibility for problem solving.

It results that an appropriate complex problem model should underline:

- System goals—allowing the definition of specific parameters to be measured, transferred, contextualized and used as information for solution evaluation.
- Available system resources, for the time horizon of problem-solving—every resource allowing specification of functionality (workflows available—as knowledge stakeholders) input/output information and triggering events.
- Problem context—external information available and, respectively, necessary.

Such a model allows, once the appropriate solution is found, to design appropriate perceptive interfaces either between interconnected systems (including human operators) or with the environment.

Perception in an ICE environment may be referred as the active process that facilitates the interpretation of the environment by integrating information and “stimuli” from the sensory system. Two processing mechanisms can be relevant in the context of an enterprise environment:

- “bottom-up” or non-aware consisting mainly of passively listening to all sensorial channels);
- “top-down” or aware consisting mainly of anticipating and selecting certain stimuli, also referred as perception control).

Relevant aspects of human brain functions and processes that can be modeled in enterprise processes and systems, include:

- Forecasting of information that is expected in a certain situation;
- Focusing on specific information (neglecting other information);
- Interpolating data with respect to an existing pattern, when real information is presenting gaps (the phenomenon of “we see what we expect to see”);
- Structuring large amounts of (sometimes possible irrelevant) information, in order to select, during the process of reasoning, the relevant one;
- Hierarchically organization of recurrent interconnected networks each providing specific functions;
- Connectivity patterns distributed among functionally specialized processes based on input-output connectivity specifications and local architecture;
- Dynamics of interfaces between functionally specialized processes;
- mechanisms allowing processing modules to incorporate adaptive changes enhancing processing system parameterization with respect to the adaptability factors of the network;
- Dynamically reconfigurable models which are incorporating functional clusters;
- Adaptive agents which connect depending on context and on the complexity of the goal;
- Interconnection process of several structural and functional modules in parallel-distributed configurations that are generating emergent behavior.

Three phases of perception can be identified, in association to the following gestalt principle: every person has a role in one’s perception process, designating a three-stage sequence:

1. Hypothesis related to perception. This will guide the selection, organization and interpretation of the stimuli.
2. Acquisition of information from the sensing system.
3. Comparison of the hypothesis with the acquired sensory information.

Reasoning is considered the (brain) capability of solving problems. The main components of the reasoning process in an ICE environment are: the problem identification, the problem categorization and the identification of the solution.

The solution identification process of a specific enterprise problem can be associated with a reasoning mechanism. Action based on the proposed solution generates feedback that can be measured and interpreted in order to generate a new perception/reasoning cycle. An generic perception/reasoning cycle that can be integrated in problem solving mechanisms of enterprise systems can include the following:

- The categorization phase is the one that determines how the following phases are performed:
- Success criteria;
- Selection (of the solving patterns—if any);
- Filtering;
- Fusion (on relevant input information);
- Planning, estimation, validation: an internal loop whose execution depends on the category of the identified problem, but whose goal is to advance towards the solution by a stepwise decomposition of the problem;
- Feedback;
- Evaluation of success (achieving the desired goal or estimation of the distance towards it).

Learning in an ICE environment can be associated to saving and retrieving a solved problem using a knowledge model. A deep learning process can also be associated to the enterprise system environment. In this process knowledge, used and validated for a given number of times may be retrieved “reflexively” i.e., without a volitional act. Rule mining and self-learning are being used with success in various industrial cases. One such a case is related to the development of a mixed-integer mathematical model to represent the direct energy consumption of machines and indirect energy consumption on a shop floor [18].

### 3.3. Intelligent System Interfaces 

An interface has various interpretations across technical literature: a connection, a composition of connections, a boundary, a linkage, an interaction between functional entities, a logical relation, a specification [19].

ISO/IEC standards (ISO/IEC 2382-1/1993) define interfaces as: “A shared boundary between two functional units, defined by various characteristics pertaining to the functions, physical signal exchanges, and other characteristics.” [20].

System Interface design can be addressed taking into consideration the following aspects:

- Characteristics of the system for which the interface is developed.
- Characteristics of the connected systems or of the environment.
- Interaction type. The type of interface can be related to different classifications: simple or complex, human- machine (H2M) or machine-machine (M2M), physical or logical, hardware, software or hybrid, internal or external.
- Interface structure.
- Interface behavior is related to behavioral patterns designed for the interface. Interface behavior describes how interfaces are used. This can be related to data handling, protocol, connection initiation procedure, synchronization, data-driven or event-driven.
- Interface monitoring.
- Interface configuration.
- Interface error handling. Some aspects relevant for error handling include data integrity, confirmation of transmission, error correction and error recovery.
- System security in relation to the interface.
- Interface control document.

When addressing the necessity of developing intelligent system interfaces, the following characteristics are relevant:

*Adaptation.* The adaptation of a system to changes in the environment or in an interconnected system needs to be addressed as both a change in the system itself but also as a change in its interfaces. In this context adaptation can become a function of the interface.

*Learning.* Machine learning techniques can be used in association with interface components. Patterns in data exchanged through the interface can emerge as a result of machine learning techniques applied at interface level. Such patterns can be used to optimize the functions of an interface. An example can be related to the amount of time the interface is used.

*Prediction.* Prediction is not only associated with intelligent systems but can become a function of the interface. Components fulfilling this functional role should be able to generate predictive models related to the environment and/or behavior of the interface.

*Optimization.* An interface can be extended with components that can monitor the function and behavior. Aggregated data from such components can be used in interface optimization processes.

When developing intelligent interfaces, the following aspects need to be addressed:

- Interface structure in accordance with system structure and behavior.
- Interface behavior in relation to the input and output data.
- Interface functionality in correlation with inputs and in accordance with the needs of the system.

## 4. Design of Systems Interfaces for the Intelligent Cyber Enterprise 

In the current section the authors propose a model for a generic perceptive systems interface as a part of an ICE architecture. In order to implement the perception function two facilitator components are proposed: a process/behavior identification component and a semantic routing component. The semantic routing component facilitates the transfer of data and information between enterprise system components. The process identification component discovers behaviors associated to the function and current use of the interface (how the interface is used) in accordance with patterns identified in the data and event streams transferred through the interface. As the interface connects system components, the process identification system analyses data in order to determine a workflow or a process associated with data passing through the interface.

### 4.1. Intelligent Cyber Enterprise Perceptive Systems Interface

The main functional aspects of a perceptive interface are showcased in the context of an ICE system model, described in this section. The proposed interface relies on components that enable its adaptation through the discovery of behavioral models based on observations of transferred information and then by selecting the appropriate behavior from a behavior repository.

**Figure 2:**
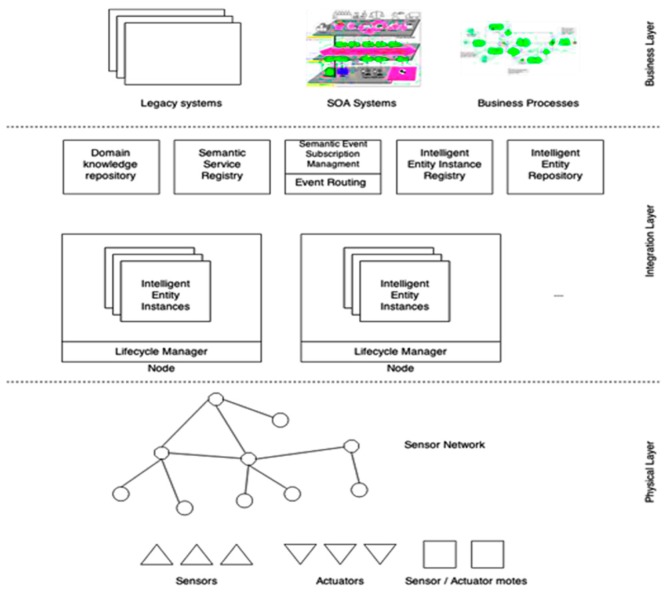
Generic ICE system interface and system model.

The ICE architecture enabling the usage of perceptive system interfaces is depicted in Figure 2 and has the following components:

*Integration Layer*—this layer is responsible to integrate data from all levels of the system and to provide the basis for the design and implementation of the cyber intelligent systems. This layer is also responsible with the integration of all nodes and layers intro a homogeneous structure that hosts the intelligent entity instances needed to manage this structure. In order to implement the integration layer, the system must have specific components in order to fulfill different roles:

*Nodes*—represent computation units that are responsible with hosting the intelligent entity instances. The entire lifecycle of a node is managed by the “lifecycle manager”. Each node must expose its own semantic interface which appears in the system as an Intelligent Entity. Through this interface, every node can receive different commands in order to check the state of every instance or to generate new events.

*Domain Knowledge Repository*—this repository is responsible with the interpretation of the system’s domain in an easy to understand machine format. The elements of the domain must be uniquely addressable so that metadata can easily target the domain’s concepts. Also, the relation between all components of a system domain must be clearly stated for the domain knowledge repository to provide reasoning capabilities and thus, to be capable to determine the context in which the needed information is processed.

*Semantic Service Repository*—this repository is responsible with the storage of the semantic description of all the services that are exposed by the intelligent entities instances. To achieve this, each service must contain metadata that refers to the system’s knowledge base. Thus, these services can be described based on existing standards (e.g., OWL-s, SAWDL, etc.).

*Semantic Event Subscription Manager/Event Routing*—this layer is responsible with the subscription of all the system’s components to the semantic events that are generated by the intelligent entities. This layer must select the exact events based on specific contexts in order to generate an event generation rule such as subscription to the events generated by sensors. The information regarding subscriptions must be sent to the “semantic event router” which is responsible to the transfer of information between the event generator and the subscriber.

*Intelligent Interface Instance Registry*—this registry is responsible with the storage of all the information that is sent through the entire system and that is deployed in the intelligent instances, thus, providing a shortcut to the services that are exposed by the intelligent entities.

*Intelligent Interface Repository*—this repository is responsible with the management of each process creation and deployment or un-deployment of the intelligent interface. On the other hand, this repository is responsible with the management of every behavior of the Intelligent Interface.

The perceptive system interface is deployed at the boundary of each intelligent entity instance and requires the following components: semantic interface, enterprise system adapters, physical adapters, lifecycle manager and behavior selection/execution component. 

**Figure 3:**
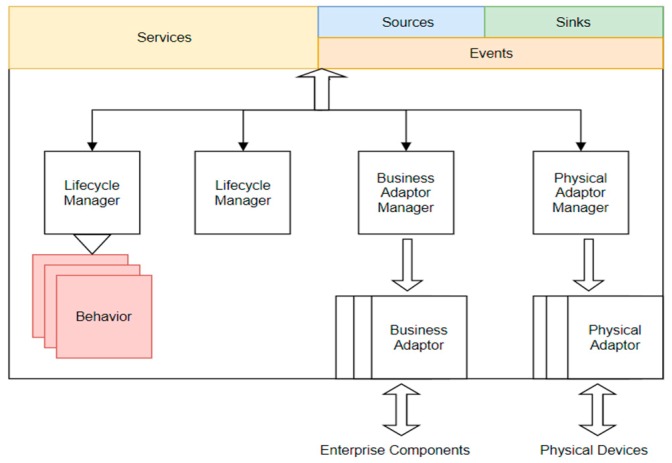
Block diagram of an intelligent entity instance.

The proposed interface (Figure 3) will allow the connection of both virtual and physical resources of an enterprise system. The use of semantic data models facilitates service and event-based interactions between the system’s components. The components of the interface: service interface, event sinks and event sources include a semantic description and the information transferred between the systems is semantically enhanced.

Enterprise system adapters are components that facilitate the connection to services, business process execution engines or service orchestration engines. Behavior selection will allow the adaptation in accordance to the process requirements. The semantic augmentation of data will facilitate data interoperability between system components:

Physical adapters—adapters for the physical devices. These adapters are designed for data acquisition and integrate sensors, sensor networks and sensing systems. These adapters can also connect actuators, actuator networks and actuating systems providing input form control systems.

Behavior Selection/Execution System. Behaviors are associated with execution engines. The selection system will facilitate the selection of the appropriate behavior, from the behavior repository, in accordance with predefined criteria.

Lifecycle manager addresses the following operations: initializing, maintenance, management of the components of the current instance and selecting behaviors or adapters. Using a semantic interface, the lifecycle manager can expose a set of services including deploy components of the instance, routing to the appropriate event source. 

### 4.2. Semantic Routing System

The ICE interfaces need to be associated with a scalable, semantically enhanced middleware solution that facilitates the integration of required resources in a distributed system. The middleware solution is characterized by the following elements [21]:

- Integration of semantics—this middleware solution manages the system’s ontology that is used to describe each component of the system and their interactions. This ontology will furthermore help in accessing resources and to the creation of the applications that are built on top of the middleware in order to provide information regarding the state of the system;
- The solution must support communication at different levels within a distributed system (e.g., service-based or event-based);
- The solution must contain all the features that are implemented in the system and thus, being capable to enable the development of robust and scalable systems.

On the other hand, the systems that are developed based on the middleware solution must have a homogeneous distributed nature, so that, within each node of the system, the middleware can have the same components and will execute the same operations. Each node of the system is determined by specific applications that, together with the distributed nature of each system built using this middleware solution, represent the basis for the creation and implementation of the ICE.

Thus, the middleware solution components are further detailed:

Components that manage the resources of each local node include: node manager and node application manager.

Components that manage the semantic layer include: semantic manager, semantic service executor, automatic event manager, distributed RDF store and the P2P overlay manager.

The P2P overlay manager supervises the P2P overlay network containing the nodes of the system. Scalable distributed systems can be developed using structured P2P overlays based on distributed hash tables (DHT). P2P overlay stores key-value pairs in the nodes of the system. The system facilitates nodes to become part and leave the network without interfering with the system’s operation. A uniform distribution of data across the network is desired in order not to generate congestions. The proposed component uses overlay to manage RDF triples and to transfer messages containing results of the query evaluation.

The semantic manager component facilitates the system components access to the system’s ontology. The ontology referring functions of the semantic manager is implemented using Apache Jena. The semantic API exposes functions that allow easier creation of individuals from the classes of the ontology including application, node, event. The semantic service executor component allows system components to invoke remote semantic services. 

The automatic event manager facilitates the event-based connection of the system components. New events will be accepted only if they are in accordance with the predefined requirements. The operation of the declarative event management system is associated with the automatic event manager component. The semantic manager component handles system’s ontology new definitions of EventSink individuals. The component identifies the corresponding event sources by using semantic queries containing properties related to the event sink descriptors.

Therefore, the proposed middleware solution must have the capability to manage all the processes of most of the applications that are deployed in the “managed applications container” managed by the node application manager. Analyzing these applications, we have identified various operations that must be exposed, bundled into the following APIs:

- Semantic API—they represent basic operations of the system’s ontology.
- Event/Quert API—they are responsible with the execution of SPARQL queries with RDFS reasoning of the distributed semantic store.
- Service API—they enable each application to call remote semantic services.

In order to achieve the best results, the middleware solution must contain a set of predefined semantic definitions that are responsible with providing a consistent implementation of communication and interoperability at different levels and thus, to support various features (e.g., remote application management) [22,23]. On the other hand, on top of these semantic definitions, each system must define its own semantic class.

### 4.3. Process Identification

The focus of this section is to detail the application of process discovery techniques on data streams in order to generate behaviors for the adaptive interface. Although most of the steps required to generate the relevant sequences of events are specific to each implementation, for some of the common ones, domain-independent solutions are investigated [12,24]:

*Event identification*—using the data transferred through the interface, which might be in continuous form, to create sequences of events relevant to the application domain. This is a domain-specific problem as the rules for extracting events from observations are dependent on the layout of the monitored environment. Internet-of-Things oriented systems can be used in relation to processing large streams of data coming from heterogeneous devices, such as employing semantically-rich representations.

*Activity recognition*—transforming sequences of “low-level” events into sequences of activities that will be represented in the resulting process model; this task is especially important given the granularity of the input data streams considered here. Solving this problem requires domain-dependent knowledge in the form of “activity models”.

*Process instance or process case identification*—this task involves associating each collected event or detected activity to one of more process instances; it should be noted that, depending on the application, this pre-processing step can be applied before or after the activity recognition one. The solutions are mostly limited to domain-specific rules, for example, based on the resources (and/or agents) involved in each process instance.

**Figure 4:**
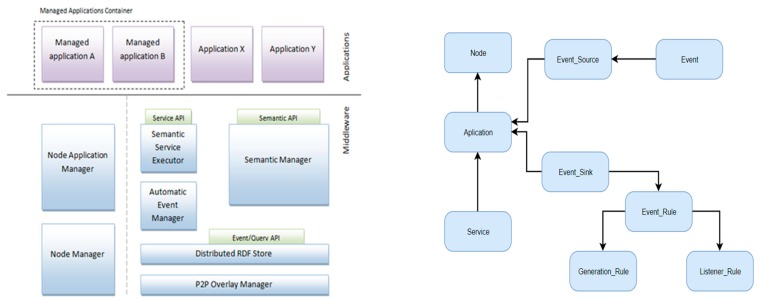
Semantic routing system and middleware ontology.

A process mining system implementation can be developed around the previously described tasks of event recognition/collection, process instance identification, action recognition and process model discovery, following an informational flow similar to that depicted in Figure 4.

**Figure 5:**
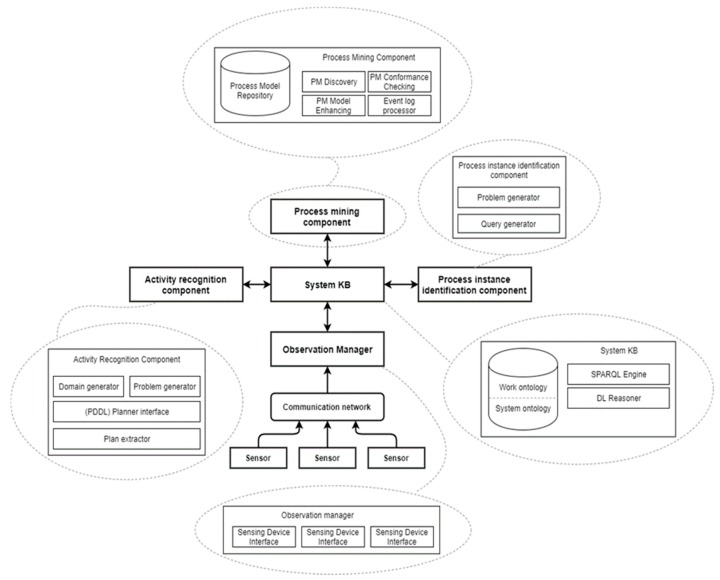
Process identification system model.

Taking into consideration several design concerns such as portability—making the system capable of handling several related application domain—and the minimization of the effort required the experts in providing the necessary system KB, a system architecture similar to the one presented in Figure 5 can be implemented—a set of major functional blocks, each corresponding to one of the previously mentioned processing tasks loosely arranged around a central data repository containing everything from raw collected to events to recognized activity sequences and process instances.

The required input from domain experts, for each processing tasks, is:

- Event identification—rules for mapping streams of observations to event sequences;
- Activity recognition—depending on the complexity of the monitored environment and the event sources, ranging from simple translation rules (for events generated by “virtual sensors” is fetched from the logs of existing workflow-management systems) to state-space action models;
- Process instance identification—the “observation manager” component handles the data acquisition and optionally the process of “semantically lifting” the observations based on a set of domain-specific transformation rules. The other components of the system each deal with one of the three information processing tasks, with the “process mining component” relying on the results generated by the other two. The “process instance identification component” can be implemented using either domain specific rules (such as a combination of keys that can uniquely identify a process case) or around a generic “event correlation” detection algorithm. The translation from the “event” to the “activity” abstraction level is performed by the “activity recognition” component. This processing step can be performed either before or after running the instance identification procedure. As mentioned in the previous section, the “high-level” activities that result in the sequences of observed events can be inferred using a schema that generates a pair consisting in a planning domain and problem, solvable using an off-the-shelf external planner.

The result of solving each planning problem will be an activity trace, from which the causal and independence relations between the activity instances can be derived. This information is subsequently used to build a workflow net. Unlike many process discovery methods that use an “event log” as input, newly proposed approaches based on Petri Net “unfoldings” accept (labelled) partial ordered sequences of events and as such offer better results from a smaller sample size in the case of processes with high concurrency (of course, having the downside that this information must be provided by an expert, or derived from another source).

## 5. Implementation of the Intelligent Cyber Enterprise System Interfaces and Event Routing System 

### 5.1. Implementation of the Event Routing System 

The proposed implementation involves the routing of data to a specific systems interface. The implementation of an event routing system facilitates the integration of new data sources such as sensors or system components without the reconfiguration of the communication network [25]. The data is routed to the appropriate source based on the semantic description. In order to perform the routing process, the following sequence of operations is proposed:

1. An application connected to a node generates a new instance of class EventSink though the semantic API.
2. The semantic RDF store is updated and the component notifies the automatic event manager (AEM) component.
3. The AEM receives the description of the sink and extracts the ListenerRule and the GenerationRule.
4. A list of event sources is obtained by the AEM calling the methods defined in Query API using the SPARQL query defined in the ListenerRule component. The AEM registers the query in as to obtain notifications when sources are available.
5. The process associated to the event routing is configured by the AEM based on the event sources register.

- The association event-node is identified based on the system ontology association of nodes with EventSource class individuals.
- Every identified node is associated by the AEM with sematic service exposes by the AEM component. The function of the semantic service is to manage the event generation process related to the requirements of the application associated to the identified EventSource. The description of the GenerationRule is correlated with the details of the new EventSink.
- The AEM creates events based on the predefined requirements of the application.

The implementation of the event routing system is based on a P2P overlay network that allows dynamic configuration of new nodes. Identified nodes are integrated in the system without interfering with the systems operation. Operations of the event routing process are distributed. The operations involved in the process of adding the node to the system are further described:

- The Node Manager component of node initializes the local components and the node manager defines the EventSources and semantic services.
- The AEM selects a sink for the event source form the applications that imposes a value change event generation. The node manager component sends the first event signaling a node start.
- Deployment events related to the new application are generated, events that can be used by the SI to update a detailed view of the system.
- The deployed application configures an EventSource. This registration will trigger a notification of the AEM component, that monitors the creation of new EventSources that correspond with the context requirements of the EventSink.
- The ARM receives the notification and configures a “value-change” event generation strategy for the registered EventSource.

### 5.2. Process Identification Based on Interface Traffic

By addressing the enterprise as a CPS, different enterprise systems can be interconnected and data collected form sensors can be aggregated. An industrial scenario was chosen involving the following event sources: position detection devices, RFID readers and contact sensors. The scenario involves human activities and the use of sensors that are present in an industrial environment but used for different purposes and by different industrial systems. The scenario is related to the movement of parts in an industrial environment. The part is transported in a container, by a worker operating a vehicle. The contact sensor detects the opening of a gate. The RFID reader identifies the tags on the vehicle and the container. The vehicle travels through three areas: AREA 1 to AREA3 and a position detection device, detects the vehicle in the specific area. 

The first step in extracting the process model involves the event identification tasks. Through the application of rules such as “An observation collected from RFID reader X of a tag Y implies that Y is currently in AREA 1”, an event sequence such as the one in Table 1 can be interpreted in relation to a specific component.

To showcase a possible solution for the activity recognition tasks using the state-based action model library mentioned previously, the movement of vehicles can be considered. The action models are designed in terms of the system’s state space. Each action definition will specify the action’s preconditions and effects as expressions based on state variables. As such, the action models will not directly refer event types, leading to the separation of the knowledge engineering tasks required in order to deploy the observation manager and activity recognition components.

As mentioned previously, the action recognition problem can be converted into a planning problem. This approach allows the reuse of existing tools developed in the field of automated planning such as PDDL editors and planners with various capabilities. For this approach, the development of a “template” planning domain and problem definitions is required. The template contains a type/object hierarchy, a set of predicates (for describing the state) and a set of action definitions.

Listing 1 details an action definition for “Transport”. Procedures can be developed to infer the type hierarchy of the planning domain from the system’s Knowledge Base.

**Listing 1.** Action definition for “Transport”

(:durative-action transport

:parameters (?v—Vehicle ?l1—Location ?l2—Location)

:duration (= ?duration 22)

:condition 

(at start (at ?v ?l1) (next_to ?l1 ?l2))

:effect

(at end 

(and

(at ?v ?l2)

(not (at ?v ?l1))

) ) )

A set of PDDL domain / problem files is created, based on the template defined for each instance of the action recognition problem. Using the information resulting from the event detection phase, the “init”, “goals” and “actions” will be augmented (Listing 2). 

**Listing 2.** Augmentation of “init”, “goals” and “actions”

(:init 

…

at 5023f674ae4d2e (event3_pred)

at 5023f674ae4d2e (not (event3_pred))

)

(:goal and(

…

(event3_constr_satisf)

))

(:action event3_constr

:precondition (and (event3_pred) (at_loc vehicle1 Area3))

:effect (and (event3_constr_satisf) (increase (total-cost) 2))

)

The schema used to constrain the trajectory, relies on the usage of timed initial literals, available since PDDL 2.2. In the proposed case, a timestamp and a “window” predicate is be enabled and the predicate is be added as a goal. This predicate is related to a new action, whose preconditions are related to an event. A conjunction of predicates is describing the change in the monitored environment’s state represented by the event, which in this case corresponds to “vehicle1”’s arriving in “Area2”. Similar constructs will be added for all the available events.

Solving the problem with these actions will allow an existing planner to instantiate planning operators (actions) capable of “explaining” (at a higher level of abstraction) the trajectory in the monitored environment’s state space, described by the collected events. For the proposed scenario (Table 1), in the case of events at t6 and t7, satisfying the goal requires predicates “event6_constr_satisf” and “event7_constr_satisf” to be true. These can only be set by adding instances of actions “event6_constr” and “event7_constr” to the plan. However, in order to satisfy the preconditions of “event7_constr”, a “transport” action instance for the areas “Zone2” and “Zone3” must be added before it.

Several post-processing steps must be applied on the resulting plan. Such steps include the removal of the special actions used to create the trajectory constraints and the detection of the concurrency relations between activities extracted.

The process instance identification step can be performed before or after the action recognition phase, based on the initial sequence of events or the results of the activity recognition stage (partially-ordered sequences of activities). This can be accomplished using either domain-specific rules or one of the semi-automated, iterative approaches mentioned in the previous section.

**Figure 6:**
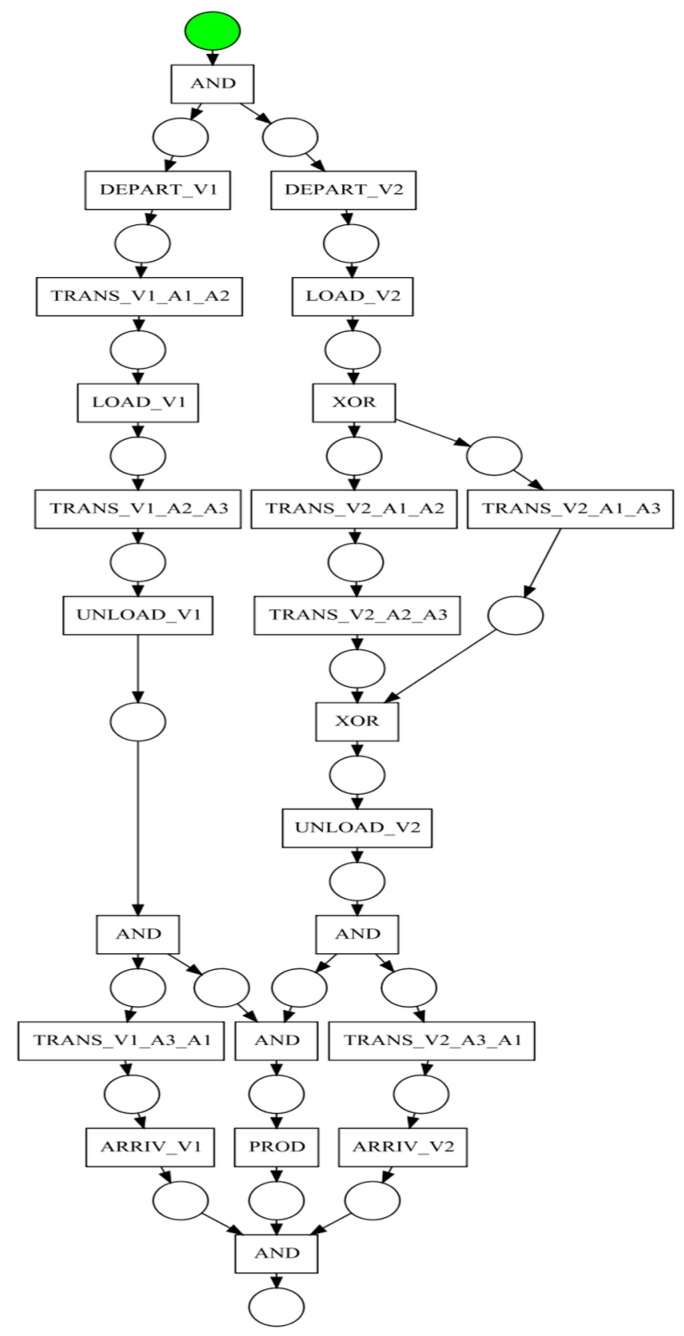
Process model of the proposed scenario.

The results obtained after the action recognition and instance identification steps can be used to compile an event log, referring high-level activities instead of low-level events. The activities can be subsequently used as the input for the process model discovery step. Given the availability of additional information regarding the concurrent execution of some of the activities (derived from the set of action models used in the recognition step), the study focused on methods capable of using this knowledge. As such, a method based on building event structures followed by folding them into Petri Nets, was used to generate a process model. An example of a process model is described in Figure 6. Some aspects of the process model such as “AND” and “XOR” blocks need to be further analyzed and modeled with the aid of a higher level Petri Net formalism. The system implementation was used to validate the proposed approach in a case study built around a simulated logistic process.

## 6. Discussion

In this article, we have explored the possibility of developing a process identification model aimed towards identification of processes associated to data transferred through a system interface. Such processes usually occur in an enterprise environment and are beyond the scope of established process management systems [26]. The identified workflows or processes are associated to the interface as interface behaviors.

The proposed model enhances the capability of a system interfaces by attaching a perception function that facilitates the interpretation of the working environment and connected systems by integrating information and “stimuli” from the sensory system [27].

Advantages of the proposed system include:

- Automated analysis of data and information transferred through a system interface;
- Pattern recognition in data and information transferred through a system interface;
- Automated identification of behaviors associated to an interface;
- Facilitation of interface redesign in accordance with the data and information transferred between systems;
- Interface operation optimization in accordance with the data and information transferred between systems;
- Enhancing system adaptation capabilities by adding the adaptation capability to the system interface.

This represents a novel approach that can provide benefits in enterprise environments adopting IoT and CPS technologies, in which a large number of events collected from sensors or system components are transferred through system interfaces [28]. The resulting interface behavior model offer meaningful insights into the behavior of the system and allows for the use of novel process mining techniques for conformance checking.

In order to address the entire complexity of the considered domain, ontologies based on DL were used. Using this method, the tasks associated to processes can be associated to reasoning problems. The proposed model demonstrates how data acquired form sensors or other system components can be analyzed and used in determining interface behaviors.

Two main categories of activity recognition problems have been analyzed in regard to the proposed: model learning based and specification base. Due to user requirements level, portability and flexibility issues only specification-based methods have been further addressed. The authors followed the main lines of research into applying and extending existing process workflow management techniques in CPS and particularly in ICE environment. Due to the benefits for industrial automation systems, the authors focused on the applications of the developed techniques in process mining in a CPS environment.

Knowledge engineering tasks are still required to develop the planning domain used in the activity recognition step and the rules that enable the identification of individual instance traces in the stream of events. An important factor for the adoption of the proposed method are the events that relate directly to state changes in the observed system. Usually process discovery techniques are used in correlation to logs of various enterprise systems.

Although the discovered process model has the same structure as the process used to generate the dataset, some performance and scalability issues related to the activity recognition step were discovered. Planning time is a relevant factor associated to the proposed method and is directly related to the increase in the system resource consumption. An “object-based” decomposition method, can be implemented in order to increase the efficiency in solving the simple planning problems and identifying “macro-operators” from existing cases. These aspects can correlate to the original domain and lead to improvements in planning time, by creating “short-cuts” in the state space and transferring them to automated planners [13,18]. Such an approach allows the augmentation of enterprise systems and their components with system interfaces capable of analyzing patterns in the transferred data and associating the patterns to workflows or processes. This approach couples the systems interface to the process associated with the actual system [12].

The event routing system facilitates the implementation of the process identification model and routes relevant data to the appropriate system interface.

The advantage proposed by the process identification model is the ability to generate process models in the absence of a pre-modeled process or to offer a basis for comparison of the existing pre-modeled process to the actual execution. The proposed approach utilizes the data already handled by the interface augmenting the function of the interface and enabling the future autonomous adaptation to process chances [12].

Further research is conducted in the area of autonomous adaptation models for systems interfaces that will allow an automatic reconfiguration of an interface in accordance with the detected changes in interface behavior and based on the selection of appropriate interface behaviors form behavior repositories [29]. An extensive discussion regarding the available translation methods for different process modelling paradigms and the association to models of interface behaviors needs to be addressed in future research.

## 7. Conclusions

A new approach to future enterprise as a complex system is proposed: intelligent cyber enterprise. The authors address the enterprise model of the intelligent cyber enterprise, describing a key component, the perceptive interface, thus proposing a method to solve the operational context adaptability problems in complex infrastructures. A behavioral model is developed, based on data and information acquired from the enterprise environment.

The proposed model integrates key functions such as processing, perception, communication, learning, pattern recognition and data mining. The proposed case studies address an enterprise environment consisting of intelligent systems-based components with a high degree of autonomy. The case studies illustrate the advantages of the proposed model in relation to complex systems behavior modeling, thus facilitating system adaptation to a dynamic working environment.

## Figures and Tables

**Table 1:** Case study observations from sensors.

Timestamp.	Device	Previous Value	Current Value
ts1	Contact_sensor_A	Closed	Open
ts2	RFID_Reader_1	-	5023f674ae4d2e
ts2	RFID_Reader_1	-	2537f674ae4d3b
ts4	Contact_sensor_B	Closed	Open
ts5	Positon_1	Area_1	Area_2
ts6	Position_1	Area_2	Area_3
ts7	Contact_2	Closed	Open
ts8	RFID_Reader_2	-	2537f674ae4d3b
ts9	RFID_Reader_2	-	5023f674ae4d2e
